# Effect of *Enterococcus faecalis* 2001 on colitis and depressive-like behavior in dextran sulfate sodium-treated mice: involvement of the brain–gut axis

**DOI:** 10.1186/s12974-019-1580-7

**Published:** 2019-10-31

**Authors:** Kohei Takahashi, Osamu Nakagawasai, Wataru Nemoto, Takayo Odaira, Wakana Sakuma, Hiroshi Onogi, Hiroaki Nishijima, Ryuji Furihata, Yukio Nemoto, Hiroyuki Iwasa, Koichi Tan-No, Takeshi Tadano

**Affiliations:** 1 Department of Pharmacology, Faculty of Pharmaceutical Sciences, Tohoku Medical and Pharmaceutical University, 4-4-1 Komatsushima, Aoba-ku, Sendai, 981-8558 Japan; 2 Department of Pharmacology, School of Pharmacy, International University of Health and Welfare, 2600-1 Kitakanemaru, Ohtawara, Tochigi 324-8501 Japan; 3 Faculty of Health Science, Tohoku Fukushi University, 1-8-1 Kunimi, Aoba-ku, Sendai, Miyagi 981-8522 Japan; 4 Department of Healthcare and Regulatory Sciences, School of Pharmacy, Showa University, 1-5-8 Hatanodai, Shinagawa-ku, Tokyo, 142-8555 Japan; 5 Department of Psychiatry, Nihon University School of Medicine, 30-1 Oyaguchi-kamicho, Itabashi-ku, Tokyo, 173-8610 Japan; 6 Kampo and Herbal Medicine Research Center, Yokohama University of Pharmacy, 601 Matanocho, Totsuka-ku, Yokohama City, Kanagawa 245-0066 Japan; 7 Nihon Berm Co, Ltd, 2-14-3 Nagatachou, Chiyoda-ku, Tokyo, 100-0014 Japan; 8 Complementary and Alternative Medicine Clinical Research and Development, Graduate School of Medicine Sciences, Kanazawa University, Kanazawa, 920-8640 Japan

**Keywords:** Antidepressant, Apoptosis, EF-2001, Inflammatory bowel disease, Neurogenesis, Neuroinflammation

## Abstract

**Background:**

Patients with inflammatory bowel disease (IBD), including those with ulcerative colitis and Crohn’s disease, have higher rates of psychiatric disorders, such as depression and anxiety; however, the mechanism of psychiatric disorder development remains unclear. Mice with IBD induced by dextran sulfate sodium (DSS) in drinking water exhibit depressive-like behavior. The presence of *Lactobacillus* in the gut microbiota is associated with major depressive disorder. Therefore, we examined whether *Enterococcus faecalis* 2001 (EF-2001), a biogenic lactic acid bacterium, prevents DSS-induced depressive-like behavior and changes in peripheral symptoms.

**Methods:**

We evaluated colon inflammation and used the tail suspension test to examine whether EF-2001 prevents IBD-like symptoms and depressive-like behavior in DSS-treated mice. The protein expression of tumor necrosis factor-α (TNF-α), interleukin-6 (IL-6), X-linked inhibitor of apoptosis protein (XIAP), and cleaved caspase-3 in the rectum and hippocampus was assessed by western blotting. Hippocampal neurogenesis, altered nuclear factor-kappa B (NFκB) p65 morphometry, and the localization of activated NFκB p65 and XIAP were examined by immunohistochemistry.

**Results:**

Treatment with 1.5% DSS for 7 days induced IBD-like pathology and depressive-like behavior, increased TNF-α and IL-6 expression in the rectum and hippocampus, activated caspase-3 in the hippocampus, and decreased hippocampal neurogenesis. Interestingly, these changes were reversed by 20-day administration of EF-2001. Further, EF-2001 administration enhanced NFκB p65 expression in the microglial cells and XIAP expression in the hippocampus of DSS-treated mice.

**Conclusion:**

EF-2001 prevented IBD-like pathology and depressive-like behavior via decreased rectal and hippocampal inflammatory cytokines and facilitated the NFκB p65/XIAP pathway in the hippocampus. Our findings suggest a close relationship between IBD and depression.

## Background

Inflammatory bowel disease (IBD), which comprises ulcerative colitis and Crohn’s disease, affects approximately 2.2 million people in Europe and 1.4 million people in the USA. Recent studies have demonstrated a connection between intestinal inflammation and changes in brain function [1]. Inflammation in the bowel is associated with alterations in the central nervous system, as revealed by the activation of tumor necrosis factor-α (TNF-α) signaling and microglia in the brain [2]. Other researchers have demonstrated that chronic experimental colitis increases anxiety behavior in mice [3]. Further, peripheral inflammation may account for at least some of the neurological and behavioral changes associated with chronic inflammatory diseases. Indeed, patients with IBD have higher rates of obsessive–compulsive disorder, panic disorder, depression, and anxiety [4–7].

A well-characterized mouse model of IBD is produced by repeated administration of dextran sulfate sodium (DSS) in drinking water [8]. DSS does not cross the blood–brain barrier because of its higher molecular weight. Epithelial cell toxicity, increased intestinal permeability, and macrophage activation are implicated in the deleterious effects of DSS. The DSS model is characterized by colonic epithelial cell lesions and acute (7–14 days after the beginning of the treatment) intestinal inflammation [9]. Repeated DSS cycling in combination with treatment with azoxymethane, a genotoxic agent, induced colitis-dependent neoplasia, generating a commonly used model for colorectal neoplasia and cancer in humans [10]. Recent studies have reported that DSS-treated rodents exhibit anxiety- and depressive-like behavior [11] and reduction of hippocampal neurogenesis [12].

Decreased adult hippocampal neurogenesis is associated with depression in rodents and humans [13–15]. Moreover, depression is associated with altered inflammation [16], which manifests due to increased inflammatory cytokine expression [17]. Pro-inflammatory cytokines also inhibit adult neurogenesis in the hippocampus [18–20]. Therefore, cytokine-induced disruption of neurogenesis might be a key link between inflammation and depression. Antidepressants enhance hippocampal neurogenesis [21] and regulate several apoptotic factors, which are involved in cell survival pathways [22]. Treatment of mood disorders, including depression and anxiety, is critically dependent on intact adult neurogenesis in the hippocampal dentate gyrus (DG) [23, 24]. Thus, the stimulation of neurogenesis and reduction of apoptosis may constitute important drug targets in the modulation of depressive symptoms [25].

*Enterococcus faecalis* 2001 (EF-2001) is a biogenic lactic acid bacterium that is used as a biological response modifier (BRM). Live *E. faecalis* 2001 can be heat-treated to produce a BRM containing high levels of β-glucan, named EF-2001. β-Glucan, one constituent of EF-2001, is a ligand for toll-like receptor 2 (TLR2) and activates nuclear factor-kappa B (NFκB) p65, which controls spontaneous apoptosis and anti-apoptotic effects. NFκB p65 activation inhibits apoptosis by increasing X-linked inhibitor of apoptosis protein (XIAP), an anti-apoptotic factor that exerts its action by regulating caspase-3 activity [26, 27]. EF-2001 can decrease serum inflammatory cytokines in a contact dermatitis model mouse [28], has anti-tumor effects [29], and protects chemical-induced colitis [30]. Therefore, we hypothesized that EF-2001 may attenuate inflammation and apoptosis in DSS-treated mice. Additionally, reports indicate that *E. faecalis* modulates inflammation in colitis models [31, 32]. However, the effect of EF-2001 on colitis-induced depression is unclear.

Taken together, we examined whether EF-2001 prevents DSS-induced depressive-like behaviors and changes in peripheral symptoms and investigated the neuroprotective molecular mechanisms underlying these effects.

## Materials and methods

All experiments were performed following approval of the Ethics Committee of Animal Experiments in Tohoku Medical and Pharmaceutical University (approval numbers: 16023 cn, 17015 cn, and 18031 cn) and according to the National Institutes of Health Guide for the Care and Use of Laboratory Animals. All efforts were made to minimize suffering and reduce the number of animals used.

### Animals

We used male ddY mice (weight, 28–32 g; Japan SLC, Shizuoka, Japan) for all experiments (total: *n* = 239; behavioral tests: *n* = 127; immunohistochemistry: *n* = 55; western blot analysis: *n* = 24; mRNA quantification: *n* = 33). Mice were housed in cages with free access to food and water under conditions of constant temperature (22 ± 2 °C) and humidity (55 ± 5%), on a 12-h light to dark cycle (lights on: 07:00–19:00).

### Compounds

Commercially available heat-treated EF-2001 was originally isolated from healthy human feces. It was supplied as a heat-killed, dried powder by Nihon BRM Co. (Tokyo, Japan). DSS (0.75%, 1.5%, or 3%; Wako Pure Chemical Industries Ltd., Osaka, Japan) and EF-2001 (250 mg/kg) were dissolved in drinking water. Mice were given drinking water containing DSS ad libitum for 7 days to induce colitis. Dexamethasone (Dex; 0.1 mg/kg; Aspen Japan, Tokyo, Japan) and imipramine (Imi; 20 mg/kg; Sigma–Aldrich, St-Louis, USA) were dissolved in saline. EF-2001 was administered orally (per os [p.o.]) from 14 days before the beginning of DSS administration until the day prior to the last DSS treatment. Dex and Imi were administered intraperitoneally (i.p.) starting on the same day as the first DSS administration until the last day of DSS treatment. The dose for each drug used was calculated from previous reports [29, 33, 34].

### Evaluation of colon inflammation

**Fig. 1:**
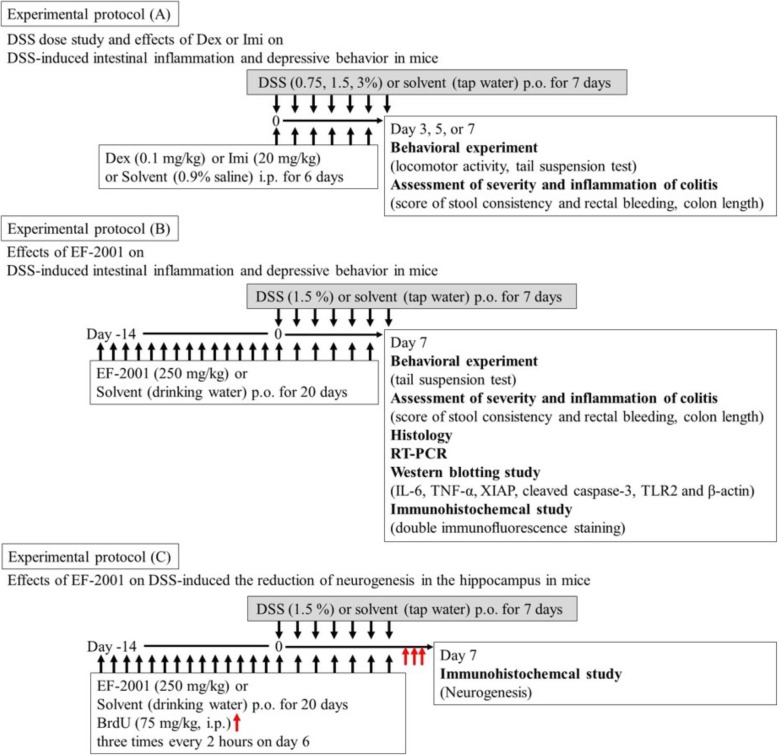
Experimental time course for the behavioral tests, assessment of inflammation, histology, western blotting, and immunohistochemical of the experimental protocol A, experimental protocol B, and experimental protocol C

This evaluation was conducted according to the experimental protocol shown in Fig. 1a, b. Disease activity index (DAI) scores are well correlated with pathological findings in a DSS-induced model of IBD [35]. DAI scores were calculated as described previously [36]. DAI is the combined score of stool consistency and bleeding, as detailed in Table 1. When mice were sacrificed, the colon length, starting above the anus to the top of the cecum, was measured. All parameters were scored on days 3, 5, and 7 during DSS treatment.

**Table 1:** Score of stool consistency and rectal bleeding

Score	Stool consistency	Rectal bleeding
1	Normal	Absent
2	Loose stool (tangible stool with high moisture content)	Visible blood stool
3	Diarrhea stool (collapsed stool with high moisture content)	Blood is attached to the anus
4	Watery diarrhea (almost intangible liquid stool)	Always bleeding from the anus

### Tail suspension test

This test was conducted according to the experimental protocol shown in Fig. 1a, b. The tail suspension test was conducted to assess depressive-like behaviors and antidepressant effects. The procedure was performed as described previously [37]. Mice were considered immobile only when they hung completely and passively motionless. Mice were suspended 30 cm above the floor by means of adhesive tape placed approximately 1 cm from the tip of the tail. The duration of time spent immobile was quantified during a test period of 10 min.

### Locomotor activity

This test was conducted according to the experimental protocol shown in Fig. 1a. The locomotor activity of mice was evaluated using the multichannel activity-counting system SUPERMEX (Muromachi Kikai Co., Tokyo, Japan). The procedure and instrument have been reported previously [38]. This instrument can monitor even minute movements in all three planes of motion (sagittal, vertical, and horizontal) as one movement, owing to its infrared sensor with multiple Fresnel lenses that can be moved close enough to the cage to capture multidirectional locomotor alterations in a single mouse. This system interprets each movement as a count. Thus, the vertical movements, such as jumping, and horizontal movements, such as walking and running, can be counted. Activity measurements were conducted between 11:00 and 15:00 during the light phase. Mice were divided into three groups (water, DSS 1.5%, and DSS 3%) and were placed in activity boxes during 15 min for adaptation. Locomotor activity was recorded for 60 min.

### Histology

The histological analysis was conducted according to the experimental protocol shown in Fig. 1b. Histology was assessed in mice following DAI and behavioral evaluations. The entire colon was collected and fixed in 4% buffered formalin for 24 h at room temperature, imbedded in paraffin, and sliced. Samples were sectioned into 5-μm slices and subjected to staining with hematoxylin and eosin (HE). Finally, sections were examined under a light microscope to evaluate the histopathologic changes in colon tissue.

### Western blotting

Western blotting was performed according to the experimental protocol shown in Fig. 1b. Mice were divided into four groups (water/water, water/EF-2001, DSS/water, and DSS/EF-2001). Mice were sacrificed by decapitation after 20 days of water or EF-2001 administration. Protein isolation and western blots were performed as described previously [37, 39]. Sectioning was performed as described previously [40, 41]. After sacrifice, the rectum of each mouse was washed in ice-cold phosphate-buffered saline (PBS). The rectal tissue, 8 mm from the edge of the cecum (side of the anus), was carefully cut into 5-mm slices on ice. The brain was removed and sectioned on ice using a mouse brain slicer (Muromachi Kikai) to produce 1-mm-thick coronal sections. To ensure precise regions, the cerebral peduncle was regarded as a landmark, and the five edge blades were anteriorly placed from this landmark. We visually confirmed the dorsal hippocampal location using Paxinos and Franklin mouse brain atlas [42]. After electrophoresis, proteins were transferred to a PVDF membrane, which was then incubated with blocking solution [10 mM Tris-HCl (pH 7.4), 100 mM NaCl, 0.01% Tween 20, and 5% skim milk] for 1 h. Next, membranes were probed with antibodies against TLR2 (1:100; Cell Signaling Technology, Danvers, USA), TNF-α (1:1000; Cell Signaling Technology), interleukin-6 (IL-6; 1:1000; Cell Signaling Technology), XIAP (1:200; Abcam Ltd., Cambridge, UK), brain-derived neurotrophic factor (BDNF; 1:100; Abcam Ltd.), and β-actin (1:1000; Cell Signaling Technology) overnight at 4 °C. Membranes were washed with blocking solution without milk and incubated with horseradish peroxidase-conjugated secondary antibody (Cell Signaling Technology) for 2 h, followed by visualization of the immunoreactive species with ECL Western Blotting Detection Reagent (Amersham Life Science, Piscataway, USA). Band densities were analyzed with ImageJ 1.43 (National Institutes of Health).

### Immunohistochemical analysis

Immunohistochemical analysis was conducted according to the experimental protocol shown in Fig. 1c. To assess neurogenesis, on day 20 after EF-2001 administration, 5-bromo-2′-deoxyuridine (BrdU; Sigma–Aldrich; 75 mg/kg, i.p.) was injected three times every 2 h after the last administration of water or EF-2001. Animals were subsequently sacrificed 24 h after the last injection. Brain samples were collected as described previously [37, 38]. The brains were cut into 40-μm sections from bregma − 2.20 to − 2.80 mm using a cryostat (MICROM HM560, Mikron Instrument, Inc., California, USA).

Frozen sections were mounted on glass slides (Matsunami Glass, Osaka, Japan). Sections were treated with HCl (2 N) at 37 °C for 30 min, followed by neutralization with sodium borate buffer (0.15 M) at room temperature twice every 10 min. After three washes every 5 min, the sections were incubated with PBS containing 1% normal goat serum (Life Technologies Corporation, Carlsbad, USA) and 0.3% Triton X-100 (PBSGT) at room temperature for 2 h. The sections were incubated overnight at 4 °C with rat anti-BrdU monoclonal antibody (1:100; Harlan SeraLab, Loughborough, UK) and mouse anti-doublecortin (DCX) monoclonal antibody (1:50; Santa Cruz Biotech, Santa Cruz, CA). Sections were washed and incubated for 2 h at room temperature with goat anti-rat IgG Alexa Fluor 568 (1:200; Molecular Probes, Eugene, USA) and goat anti-mouse IgG Alexa Fluor 488 (1:200; Molecular Probes) with PBSGT. DAPI was used to identify the nuclei. Finally, sections were washed and coverslipped with Dako fluorescence mounting medium (Dako, Carpinteria, USA). Labeled sections were analyzed using a confocal laser-scanning microscope (A1Rsi; Nikon, Tokyo, Japan). Eight sections per mouse were used, and two images (left and right hemisphere, 640 × 640 μm) of the DG region of the hippocampus were obtained from each section. To assess neurogenesis, we counted the number of BrdU^+^/DCX^+^ cells in the DG. A mean number of eight images were analyzed for each mouse, and each group contained 6–9 mice.

### Double immunofluorescence staining

Immunofluorescence analysis was conducted according to the experimental protocol shown in Fig. 1b. The brain samples were collected as described previously [43, 44]. The sections were incubated overnight at 4 °C with rabbit anti-TLR2 (1:100; Cell Signaling Technology), rabbit anti-NFκB p65 (1:500; Cell Signaling Technology), rabbit anti-XIAP (1:200; Abcam Ltd.), mouse anti-DCX monoclonal antibody (1:50; Santa Cruz Biotech), mouse anti-neuronal nuclear antigen (NeuN; 1:500; Millipore Corporation), rabbit anti-ionized calcium-binding adaptor molecule 1 (Iba1; 1:200; Wako Pure Chemical Industries Ltd., Osaka, Japan), and mouse anti-glial fibrillary acidic protein (GFAP; 1:200; Millipore Corporation) antibodies. When double labeling was performed using two primary antibodies from different host species (rabbit and mouse), sections were washed and incubated for 2 h at room temperature with goat anti-rabbit IgG Alexa Fluor 568 (1:200; Molecular Probes) and goat anti-mouse IgG Alexa Fluor 488 (1:200; Molecular Probes) in PBSGT. When double labeling was performed using two primary antibodies from the same host species (rabbit anti-TLR2, rabbit anti-NFκB p65, rabbit anti-XIAP, and rabbit anti-Iba1 antibodies), the detection of each antigen was performed sequentially and labeled goat anti-rabbit IgG Alexa Fluor 488 AffiniPure Fab fragments (Jackson ImmunoResearch Laboratories, USA), instead of whole antibodies, were used in the first detection (Iba1). The immunohistochemical staining with two primary antibodies from the same host species was carried out as described previously in detail [45]. Immunofluorescent images were analyzed with a confocal laser-scanning microscope (A1Rsi; Nikon).

### Neuromorphometrical study

Morphometric assessment of the brain was conducted according to the experimental protocol shown in Fig. 1b. The brain samples were collected as described previously [37, 38]. The sections were incubated overnight at 4 °C with rabbit anti-NFκB p65 antibody (1:500; Cell Signaling Technology). Sections were washed and incubated for 2 h at room temperature with goat anti-rabbit IgG Alexa Fluor 568 (1:200; Molecular Probes) in PBSGT. We observed alterations in activation of NFκB p65-positive cells in the hippocampal DG area with a confocal laser-scanning microscope. We then evaluated the activation of NFκB p65-positive cells by observing translocation to cell nuclei.

### Reverse transcription polymerase chain reaction (RT-PCR)

RT-PCR was performed according to the experimental protocol shown in Fig. 1b. Total RNA was isolated from the rectum and hippocampus of mice using TRI Reagent according to the manufacturer’s protocol. Total RNA was reverse transcribed using ReverTra Ace and oligo (dT) primers. PCR was conducted using the following primer sequences: IL-6 sense primer 5′-AGGAGTGGCTAAGGACCAAGA-3′ and antisense primer 5′-CATAACGCACTAGGTTTGCCG-3′, TNF-α sense primer 5′-GGCAGGTCTACTTTGGAGTCATTGC-3′ and antisense primer 5′-ACATTCGAGGCTCCAGTGAATTCGG-3′, and TATA- binding protein (TBP) sense primer 5′-ACCGTGAATCTTGGCTGTAAAC-3′ and antisense primer 5′-GCAGCAAATCGCTTGGGATTA-3′. For quantification of mRNA expression, real-time PCR was carried out in a 20-μl solution containing Go Taq quantitative PCR Master mix (10 μl), RT template (2 μl), water (7 μl), and primers (1 μl) using the StepOnePlus Real-Time PCR System (Applied Biosystems, California, USA). The amount of each PCR product was normalized to TBP.

### Statistical analysis

Normality and homoscedasticity assumptions were verified before the use of any parametric tests (Shapiro–Wilk normality test and equality of variances *F* test). Results are expressed as mean ± standard error of the mean (SEM). The significance of differences was determined by the Student’s *t* test for two-group comparisons or by one or two-way analysis of variance (ANOVA), followed by Tukey–Kramer tests for multigroup comparisons using GraphPad Prism 7 (GraphPad Software, California, USA) and StatView 5.0 (HULINKS, Tokyo, Japan). For the DAI scores, statistical significance of differences was assessed with a non-parametric Mann–Whitney test for two-group comparisons or Kruskal–Wallis test followed by Steel’s test for multigroup comparisons. In some cases, when a main effect was significant without interaction effect, we did an exploratory and limited pairwise post hoc comparison consistent with our a priori hypothesis. Results were considered statistically significant if *p* < 0.05.

## Results

### Concentration-dependent effect of DSS on DAI scores, colon length, immobility time, and locomotor activity in mice

**Fig. 2:**
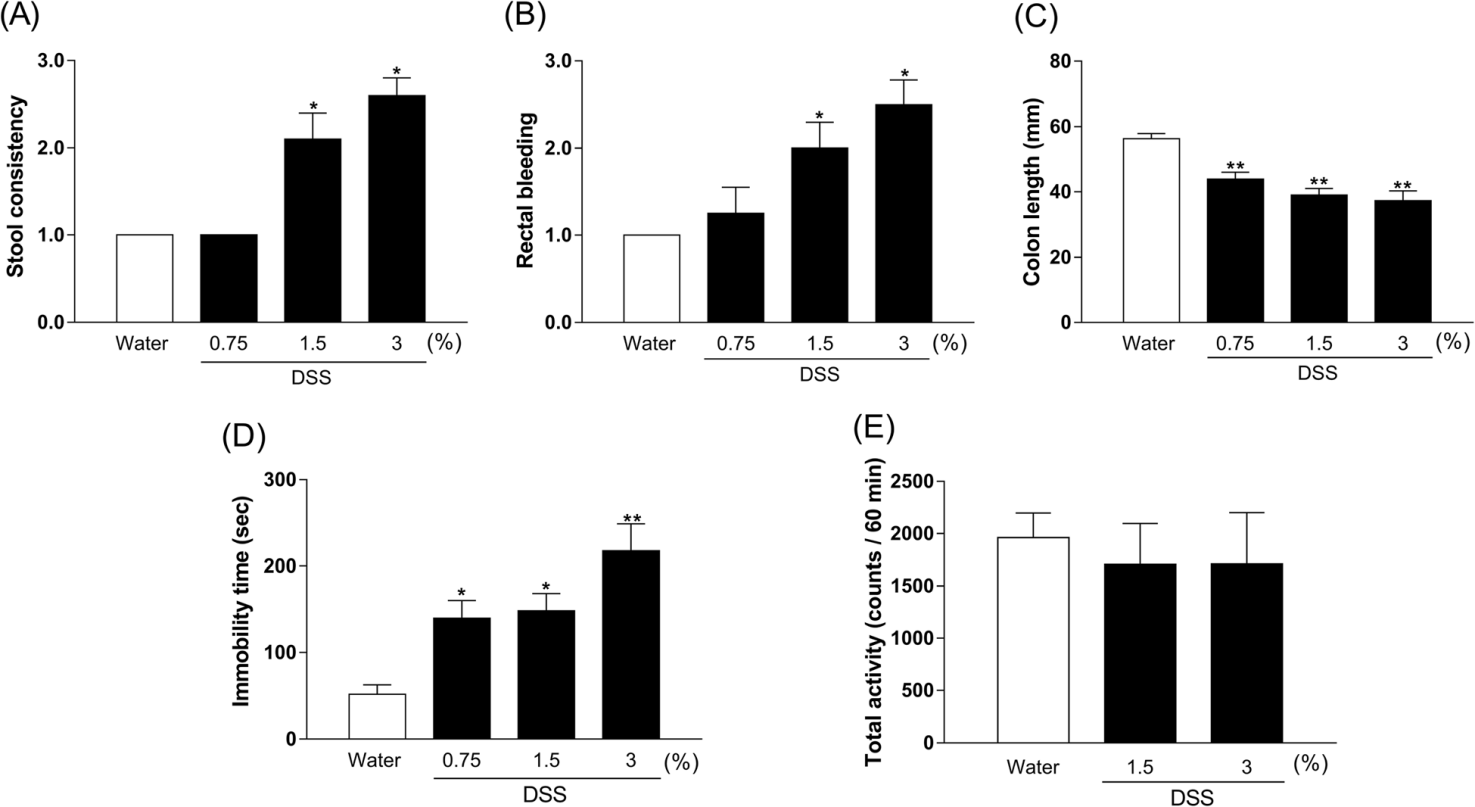
Changes in stool consistency (**a**), rectal bleeding (**b**), colon length (**c**), immobility time (**d**), and locomotor activity (**e**) in dextran sulfate sodium (DSS)-treated mice at day 7. Bars represent means ± standard error of mean (SEM). *p < 0.05 and **p < 0.01 vs. water group (*n* = 6–12 per group)

DAI scores of both stool consistency and rectal bleeding in DSS-treated mice (1.5% and 3%) were significantly increased compared with those in the control group [Fig. 2a, b, Kruskal–Wallis test, stool consistency: *p* < 0.0001, rectal bleeding: *p* < 0.0001]. The colon length in DSS-treated mice (0.75%: *p* = 0.0007, 1.5%: *p* < 0.0001, and 3%: *p* < 0.0001) was significantly shorter than in control mice [Fig. 2c, one-way ANOVA, *F* (3, 41) = 16.2, *p* < 0.0001]. There was a significantly prolonged duration of immobility in DSS-treated mice (0.75%: *p* = 0.0337, 1.5%: *p* = 0.0165, and 3%: *p* < 0.0001) compared with controls in the tail suspension test [Fig. 2d, one-way ANOVA, *F* (3, 44) = 9.626, *p* < 0.0001]. Furthermore, DSS (1.5% and 3%) did not affect locomotor activity in mice [Fig. 2e, one-way ANOVA, *F* (2, 27) = 0.142, *p* = 0.8683].

We observed that DSS 3% caused the death of a few mice (data not shown). Based on these results, DSS 1.5% was the appropriate dose to investigate in the IBD model with depression.

### Time-dependent effects of DSS on DAI scores, colon length, and immobility time in mice

**Fig. 3:**
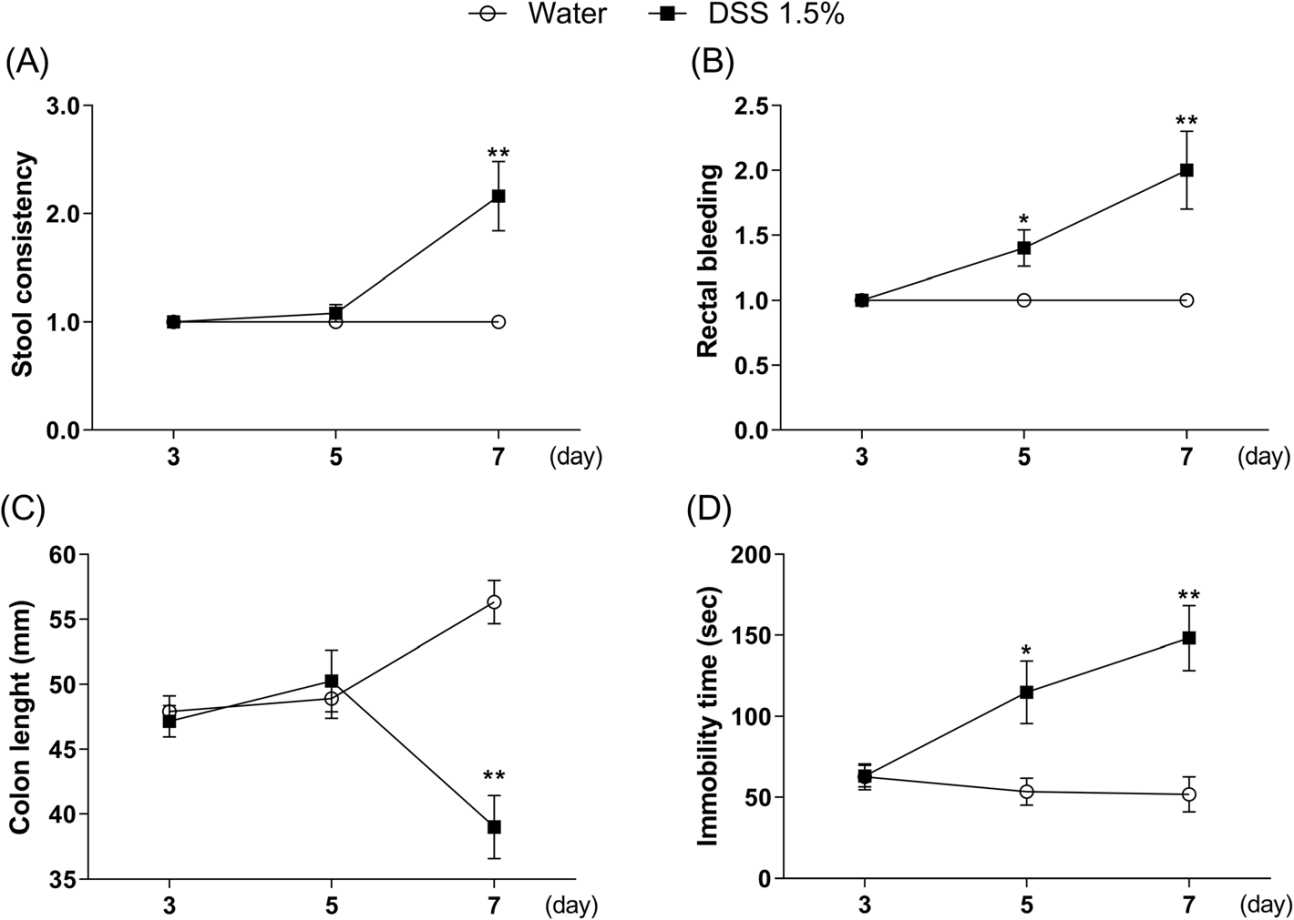
Time-course of stool consistency (**a**), rectal bleeding (**b**), colon length (**c**), and immobility time (**d**) in dextran sulfate sodium (DSS; 1.5%)-treated mice at days 3, 5, and 7. Bars represent means ± standard error of mean (SEM). *p < 0.05 and **p < 0.01 vs. water group (*n* = 11–12 per group)

As shown in Fig. 3, diarrhea and shortened colon length were observed on day 7 of DSS treatment, but not on days 3 and 5 [Fig. 3a, Mann–Whitney test, day 3: *p* > 0.9999, day 5: *p* = 0.3173, day 7: *p* = 0.0009; Fig. 3c, two-way ANOVA, group: *F* (1, 65) = 14.51, *p* = 0.0003, time: *F* (2, 65) = 0.8248, *p* = 0.4429, group × time: *F* (2, 65) = 16.31, *p* < 0.0001]. In contrast, bloody stool and prolonged duration of immobility were observed on days 5 and 7 of DSS treatment, but not on day 3 [Fig. 3b, Mann–Whitney test, day 3: *p* > 0.9999, day 5: *p* = 0.0139, day 7: *p* = 0.0008; Fig. 3d, two-way ANOVA, group: *F* (1, 66) = 23.4, *p* < 0.0001, time: *F* (2, 66) = 3.874, *p* = 0.0257, group × time: *F* (2, 66) = 6.591, *p* = 0.0025]. Based on these results, day 7 after the beginning of DSS treatment was the best time point to investigate changes in the IBD model with depression.

### Effects of Imi, Dex, or EF-2001 on DAI scores, colon length, and immobility time in DSS-treated mice

**Fig. 4:**
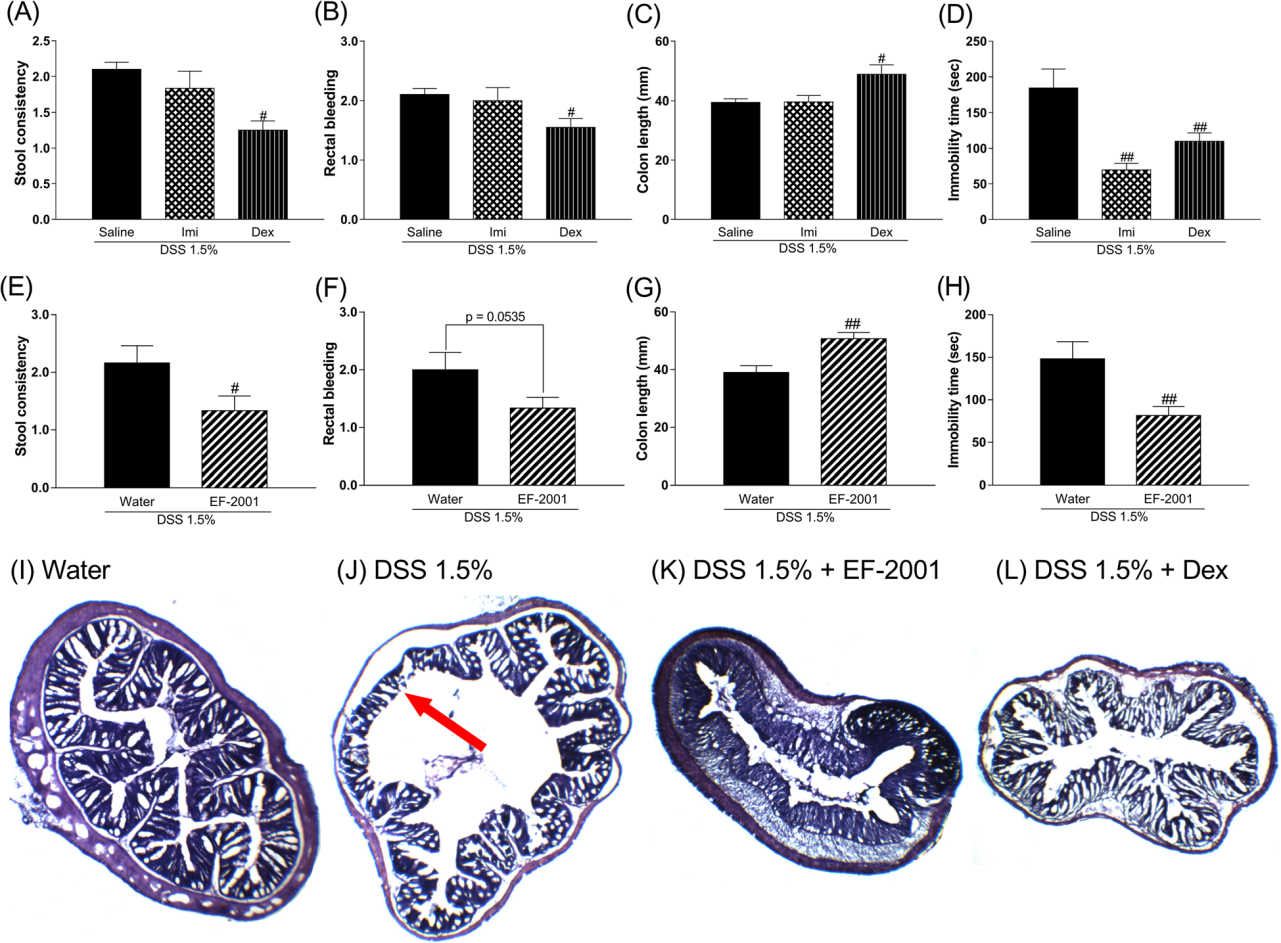
Effect of subchronic dexamethasone (Dex) or imipramine (Imi) treatment or chronic *Enterococcus faecalis* 2001 (EF-2001) treatment on stool consistency (**a**, **e**), rectal bleeding (**b**, **f**), colon length (**c**, **g**), and immobility time (**d**, **h**) in dextran sulfate sodium (DSS)-treated mice. Effect of EF-2001 and Dex on histopathologic changes in colon tissue in DSS-induced colitis. Colon tissue of the control (**i**), DSS 1.5% (**j**), DSS 1.5% + EF-2001 (**k**), and DSS 1.5% + Dex (**l**) groups. The red arrow indicates thinning of the mucous membrane. Bars represent means ± standard error of mean (SEM). ^#^*p* < 0.05 and ^##^*p* < 0.01 vs. vehicle-treated DSS group (*n* = 10–12 per group)

We investigated the effects of Imi, Dex, or EF-2001 on DSS-induced changes in mice. Imi reversed the DSS-induced prolonged duration of immobility time, while other changes were not affected. In contrast, Dex and EF-2001 prevented DSS-induced diarrhea, bloody stool (Dex showed a tendency toward prevention of bloody stool), and colon atrophy. Further, it reversed the prolonged duration of immobility time [Kruskal–Wallis test, Fig. 4a, stool consistency: *p* = 0.0012; Fig. 4b, rectal bleeding: *p* = 0.0181; Fig. 4c, one-way ANOVA: *F* (2, 31) = 5.089, *p* = 0.0123; Fig. 4d, *F* (2, 31) = 12.17, *p* = 0.0001; Mann–Whitney test, Fig. 4e, stool consistency: *p* = 0.0231; Fig. 4f, rectal bleeding: *p* = 0.0535; Student’s *t* test, Fig. 4g, colon length: *t* (22) = 3.632, *p* = 0.0015; Fig. 4h, immobility time: *t* (22) = 2.939, *p* = 0.0076]. In the histological study, EF-2001 prevented DSS-induced colon erosion similar to treatment with Dex (Fig. 4i–l).

### Effect of EF-2001 on TNF-α and IL-6 levels in the rectum and hippocampus of DSS-treated mice

**Fig. 5:**
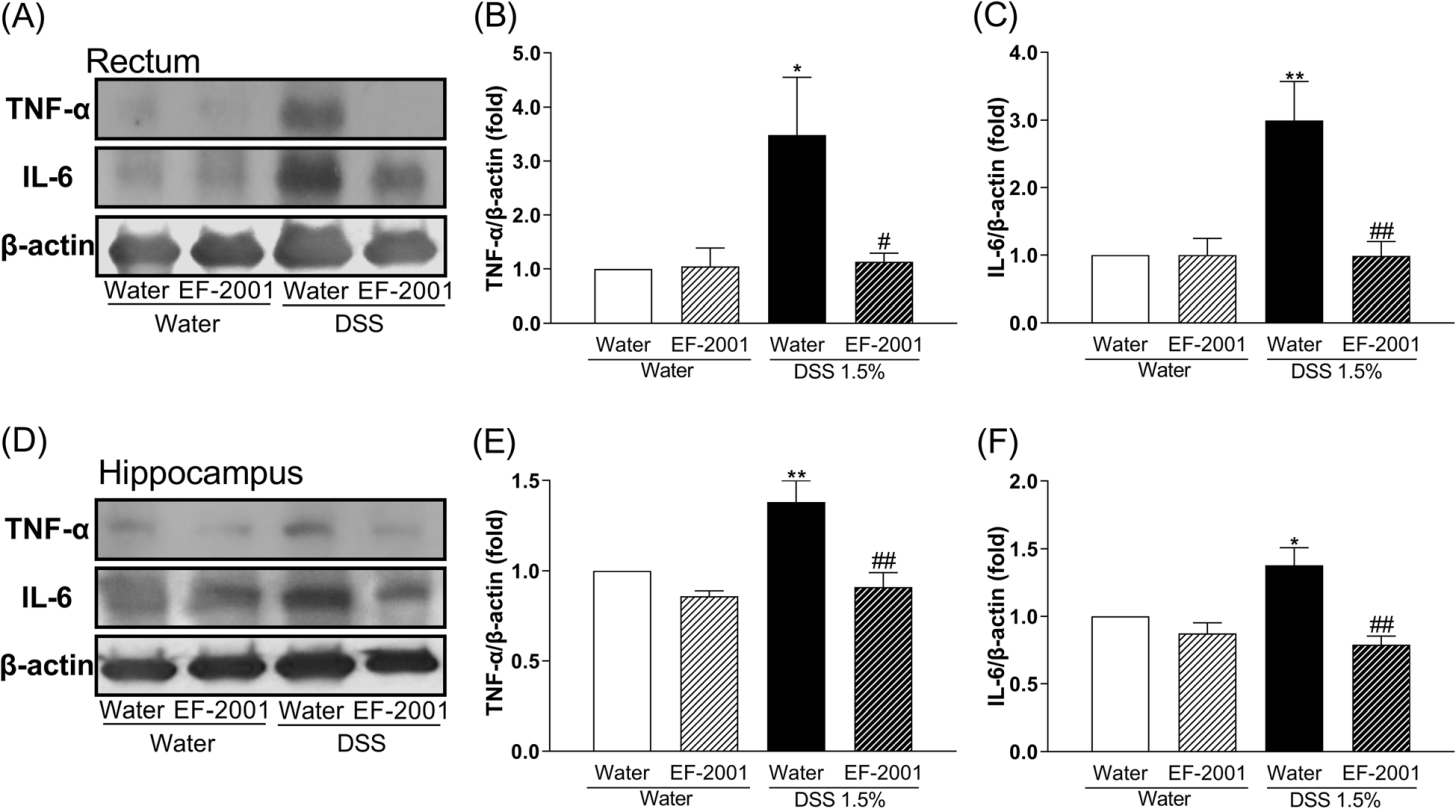
Altered levels of TNF-α and IL-6 in the rectum and hippocampus after *Enterococcus faecalis* 2001 (EF-2001) administration. **a** Representative immunoblots probed with antibodies against rectal TNF-α, IL-6, and β-actin, as indicated. **b**, **c** Quantification of normalized values of TNF-α and IL-6 levels with β-actin in the rectum. **d** Representative immunoblots probed with antibodies against hippocampal TNF-α, IL-6, and β-actin, as indicated. **e**, **f** Quantification of normalized values of TNF-α and IL-6 levels with β-actin in the hippocampus. Bars represent means ± standard error of mean (SEM). *p < 0.05 and **p < 0.01 vs. vehicle-treated water group. ^#^*p* < 0.05 and ^##^*p* < 0.01 vs. vehicle-treated DSS group (*n* = 4–7 per group)

As shown in Fig. 5, the immunocontent of TNF-α and IL-6 in the rectum and hippocampus of DSS-treated mice was significantly increased compared with controls. Interestingly, these changes were reversed by treatment with EF-2001 [two-way ANOVA, Fig. 5b, group: *F* (1, 17) = 10.01, *p* = 0.0057, treatment: *F (*1, 17) = 10.26, *p* = 0.0052, group × treatment: *F* (1, 17) = 10.27, *p* = 0.0052; Fig. 5c, group: *F* (1, 14) = 6.676, *p* = 0.0216, treatment: *F* (1, 14) = 5.352, *p* = 0.0364, group × treatment: *F* (1, 14) = 5.813, *p* = 0.0302; Fig. 5e, group: *F* (1, 22) = 3.273, *p* = 0.0841, treatment: *F* (1, 22) = 19.13, *p* = 0.0002, group × treatment: *F* (1, 22) = 8.049, *p* = 0.0096; Fig. 5f, group: *F* (1, 22) = 8.157, *p* = 0.0092, treatment: *F* (1, 22) = 16.3, *p* = 0.0006, group × treatment: *F* (1, 22) = 4.728, *p* = 0.0407].

### Effect of EF-2001 on TNF-α and IL-6 mRNA levels in the hippocampus of DSS-treated mice

**Fig. 6:**
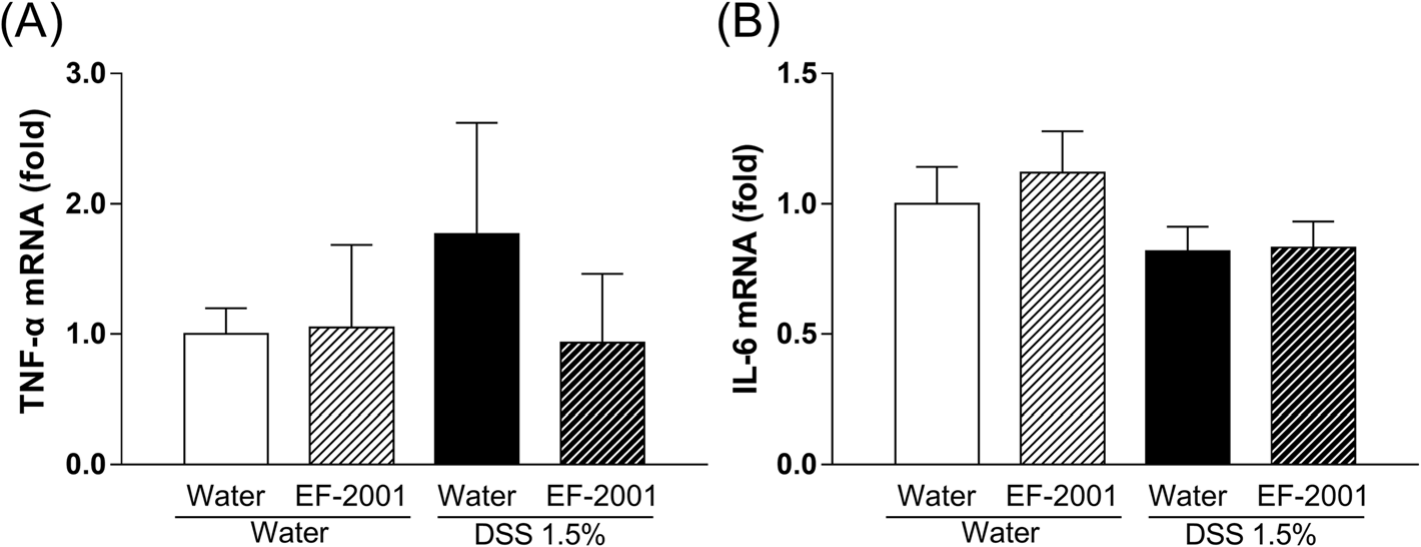
Hippocampal expression of TNF-α (**a**) and IL-6 (**b**) mRNA in dextran sulfate sodium-treated mice. Bars represent means ± standard error of mean (SEM) (*n* = 3–9 per group)

We investigated the changes in the expression of TNF-α and IL-6 mRNA levels in the hippocampus. The hippocampal TNF-α and IL-6 mRNA levels in DSS-treated mice did not change as compared to those in control mice [Fig. 6a, two-way ANOVA, group: *F* (1, 17) = 0.1801, *p* = 0.6766, treatment: *F* (1, 17) = 0.2614, *p* = 0.6157, group × treatment: *F* (1, 17) = 0.3334, *p* = 0.5713; Fig. 6b, group: *F* (1, 29) = 3.335, *p* = 0.0781, treatment: *F* (1, 29) = 0.2765, *p* = 0.6030, group × treatment: *F* (1, 29) = 0.1689, *p* = 0.6841].

### Effect of EF-2001 on reduced neurogenesis in the hippocampal DG of DSS-treated mice

**Fig. 7:**
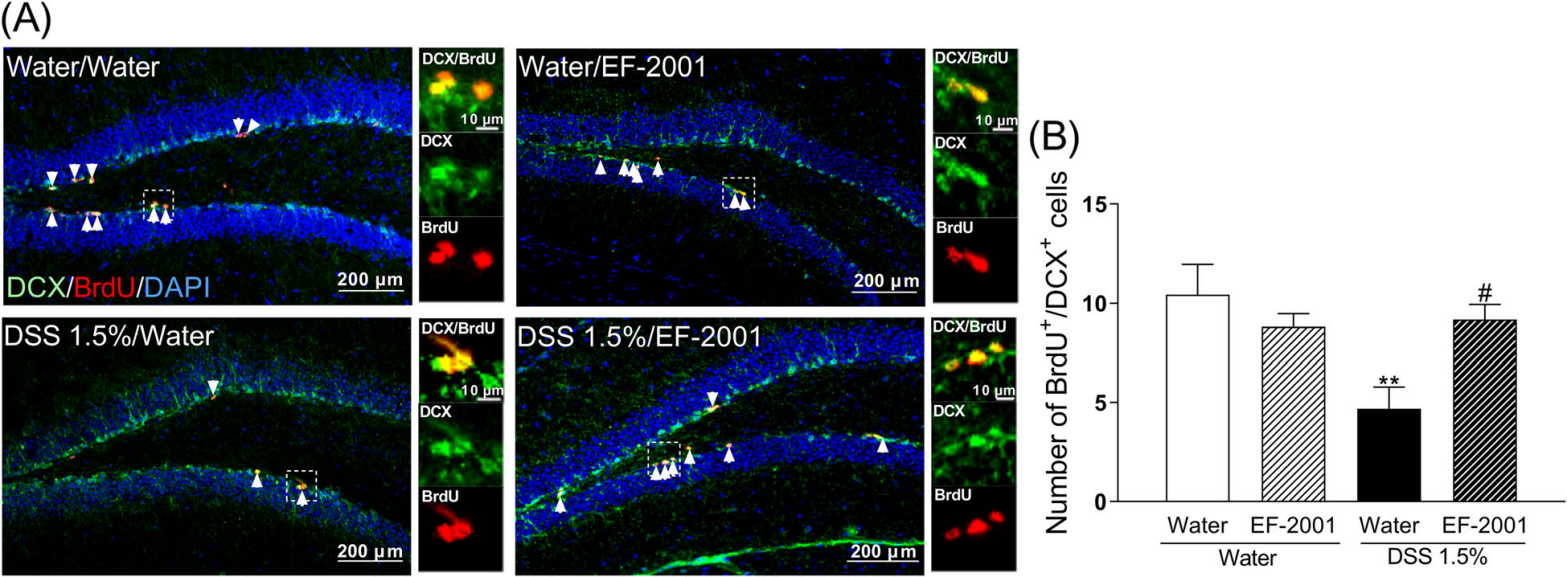
Influence of *Enterococcus faecalis* 2001 (EF-2001) on hippocampal neurogenesis in dextran sulfate sodium (DSS)-treated mice. Microscopy images of BrdU (red), DAPI (blue), and DCX (green) immunostaining in the dentate gyrus region of the hippocampus (**a**). Arrows indicate BrdU/DCX double-positive cells. Quantitative analysis of the number of BrdU/DCX double-positive cells in control and DSS-treated mice after administration of vehicle or EF-2001 (**b**). Bars represent means ± standard error of mean (SEM). **p < 0.01 vs. vehicle-treated water group. ^#^*p* < 0.05 vs. vehicle-treated DSS group (*n* = 6–9 per group)

To determine any change in hippocampal neurogenesis in DSS-treated mice, animals were injected with BrdU. The incorporation of BrdU indicates that cells were replicating at the time of the BrdU injection. Further, anti-DCX staining was used to identify immature neurons in the DG. DSS-treated mice had a significantly lower number of BrdU^+^/DCX^+^ cells compared with the control group, which was reversed by administration of EF-2001 [Fig. 7b, two-way ANOVA, group: *F* (1, 27) = 5.927, *p* = 0.0218, treatment: *F* (1, 27) = 1.663, *p* = 0.2082, group × treatment: *F* (1, 27) = 7.613, *p* = 0.0103].

### NFκB p65-positive astrocytes and microglia in the hippocampal DG after treatment with EF-2001

**Fig. 8:**
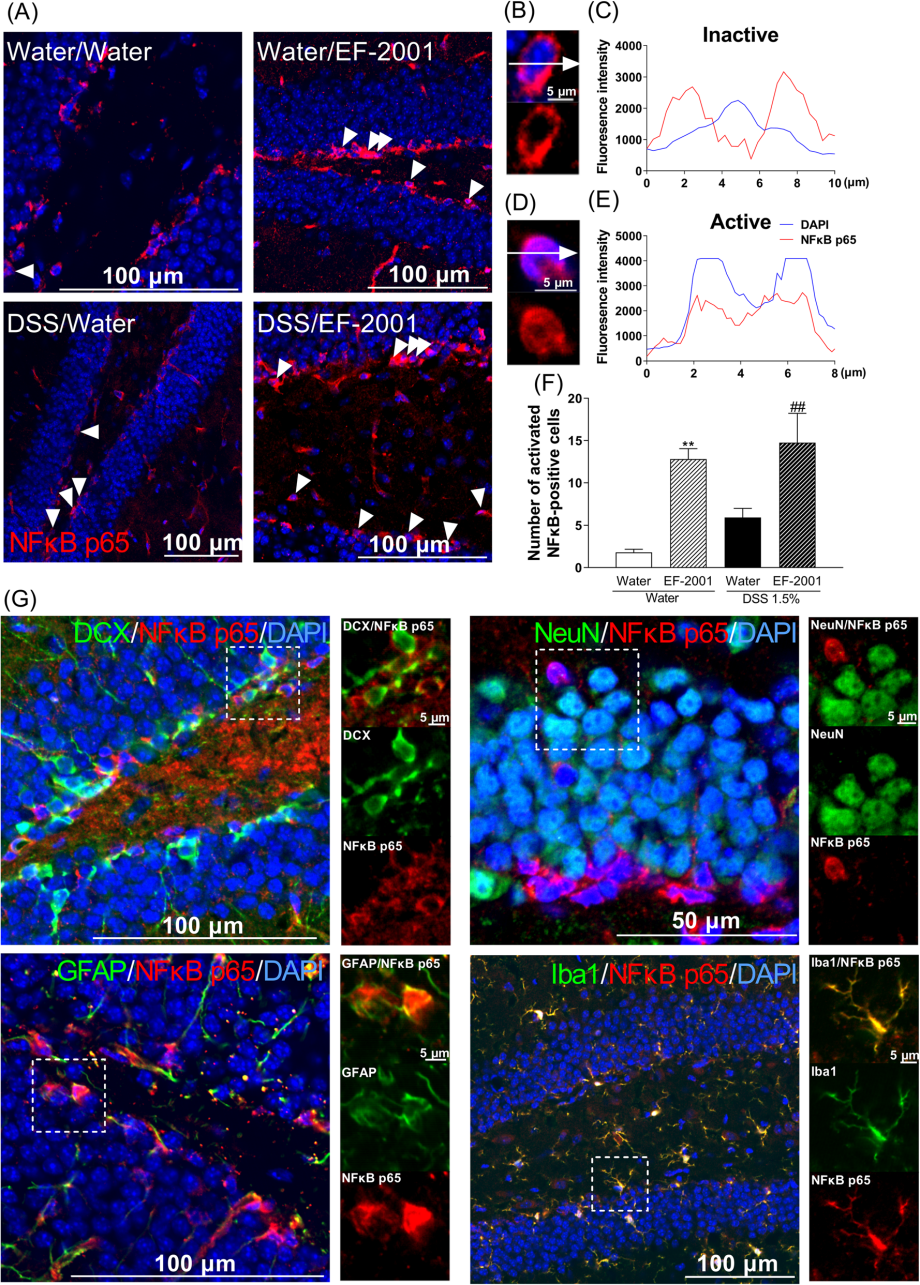
Influence of *Enterococcus faecalis* 2001 (EF-2001) on hippocampal activation of NFκB p65-positive cells in dextran sulfate sodium (DSS)-treated mice. Microscopy images of NFκB p65 (red) and DAPI (blue) immunostaining in the dentate gyrus region of the hippocampus (**a**). The fluorescence intensity profile of DAPI (blue line) and NFκB p65 (red line) in the immunostaining indicated by the white dashed lines in the inactive NFκB p65 (**b**, **c**) and active NFκB p65 (**d**, **e**). Quantitative analysis of the number of activated NFκB p65-positive cells in control and DSS-treated mice after administration of vehicle or EF-2001 (**f**). Activated NFκB p65 is expressed in astrocytes and microglia in the hippocampus of DSS-treated mice treated with EF-2001. Microscopy images of NFκB p65 (red), DAPI (blue), and DCX, NeuN, GFAP, or Iba1 (green) immunostaining in the dentate gyrus region of the hippocampus (**g**). The boxed area is shown in higher magnification. Bars represent means ± standard error of mean (SEM). **p < 0.01 vs. vehicle-treated water group. ^##^*p* < 0.01 vs. vehicle-treated DSS group (*n* = 4–5 per group)

NFκB p65 may control spontaneous apoptosis, anti-apoptotic gene expression, and translocation to cell nuclei during activation. Two-way ANOVA showed significant main effects for the treatment factor but not an interaction [Fig. 8f, two-way ANOVA, group: *F* (1, 15) = 3.237, *p* = 0.0922, treatment: *F* (1, 15) = 34.35, *p* < 0.0001, group × treatment: *F* (1, 15) = 0.4169, *p* = 0.5282]. Therefore, we focused our analysis on the effects of treatment. EF-2001 treatment significantly increased activation of NFκB p65-positive cells in the DG compared with the water-treated group. To determine which cell types were involved, dual immunofluorescence staining for NFκB p65 was performed in conjunction with cell-specific markers, such as DCX, NeuN (a marker for mature neurons), GFAP (an astrocyte marker), and Iba1 (a microglia marker). Activated NFκB p65-positive cells were identified as astrocytes and microglia (Fig. 8g).

### Effect of EF-2001 on TLR2 levels in the hippocampus of DSS-treated mice

**Fig. 9:**
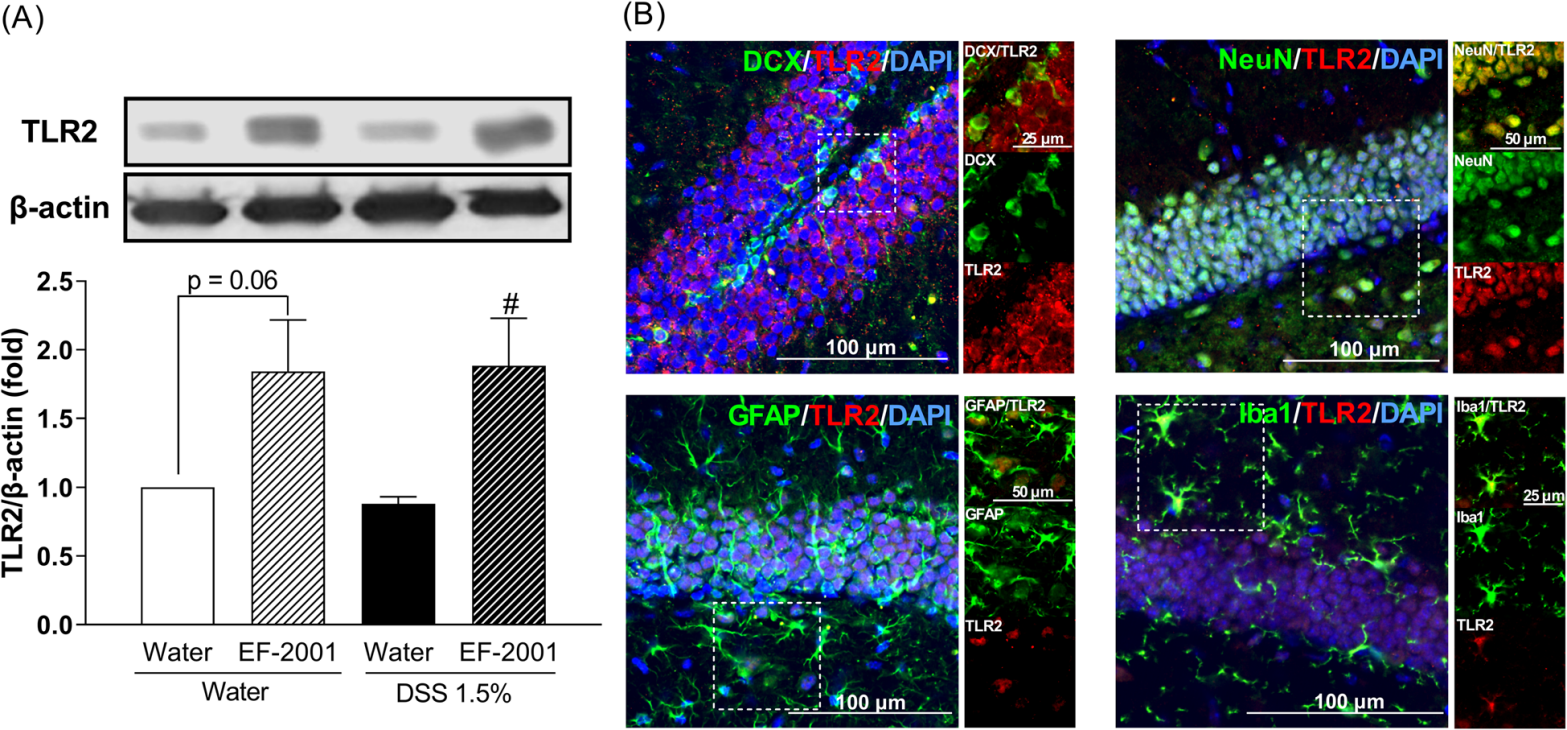
Altered TLR2 protein level in the hippocampus after *Enterococcus faecalis* 2001 (EF-2001) administration. **a** TLR2 is expressed in all cell types in the hippocampus of dextran sulfate sodium (DSS)-treated mice administered with EF-2001. Fluorescence microscopy images of TLR2 (red); DAPI (blue); and DCX, NeuN, GFAP, or Iba1 (green) immunostaining in the dentate gyrus region of the hippocampus. **b** The insets (boxed area) are images of higher magnification. Bars represent means ± standard error of mean (SEM). ^#^*p* < 0.05 vs. vehicle-treated DSS group (*n* = 4–6 per group)

Two-way ANOVA showed statistical significance for the main effects of treatment [*F* (1, 14) = 16.49, *p* = 0.0012] but no significance for the TLR2 interaction [group × treatment: *F* (1, 14) = 0.13, *p* = 0.7239] (Fig. 9a). Thus, we focused our analysis on the major effects of EF-2001 administration. EF-2001 significantly increased TLR2 expression in the hippocampus of DSS-treated mice. Moreover, to identify the cell types involved in the production of TLR2, dual immunofluorescence staining was performed for the localization of TLR2 and cell-specific markers, such as DCX, NeuN, GFAP, and Iba1. TLR2 was localized in all assessed cell types (Fig. 9b).

### Effect of EF-2001 on the enhancement of neuroinflammation and apoptosis in the hippocampus of DSS-treated mice

**Fig. 10:**
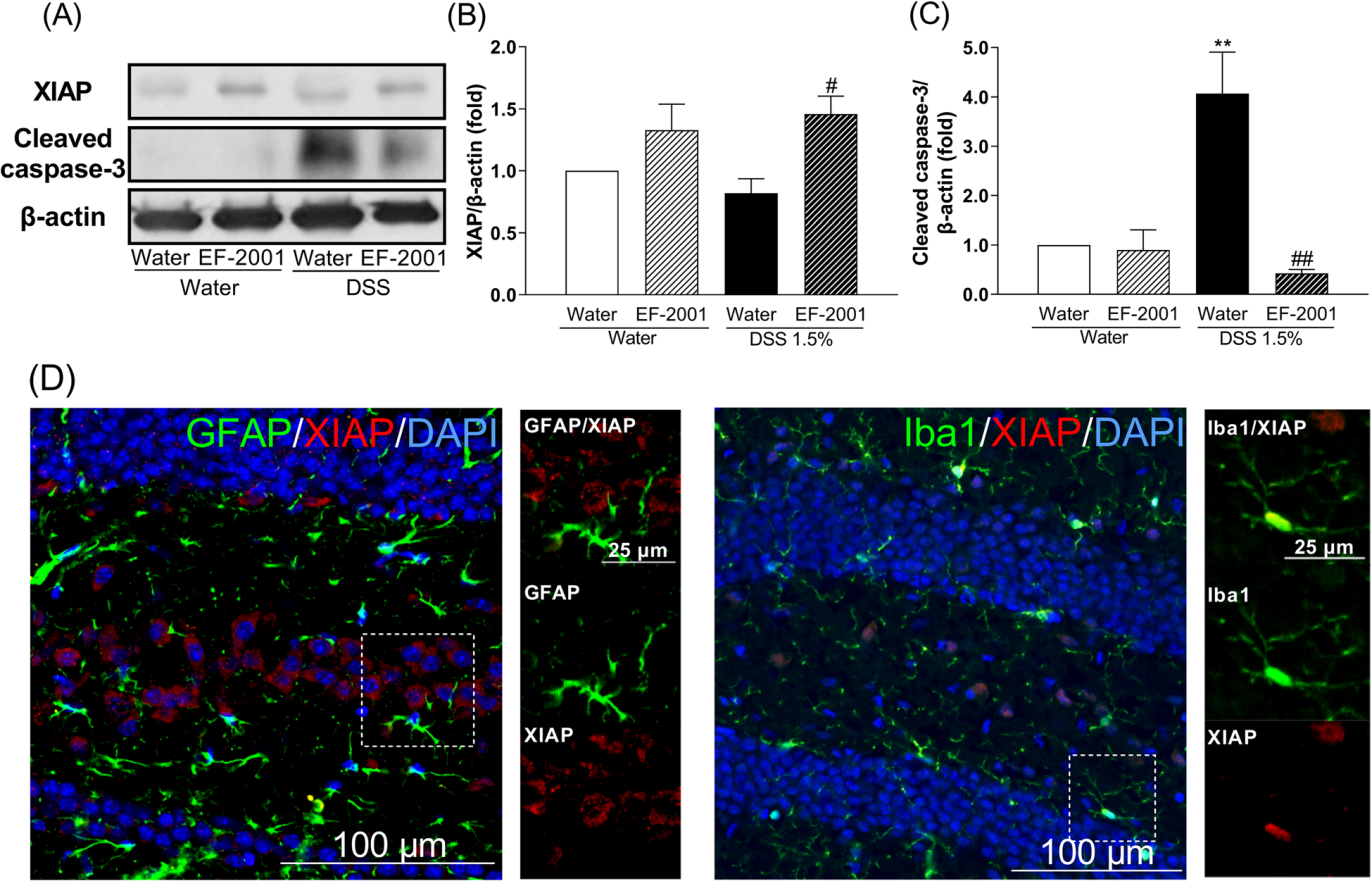
Altered levels of XIAP and cleaved caspase-3 in the hippocampus after *Enterococcus faecalis* 2001 (EF-2001) administration. **a** Representative immunoblots probed with antibodies against hippocampal XIAP, cleaved caspase-3, and β-actin, as indicated. **b**, **c** Quantification of normalized values of XIAP and cleaved caspase-3 levels with β-actin in the hippocampus. XIAP is expressed in microglia in the hippocampus of dextran sulfate sodium (DSS)-treated mice treated with EF-2001. Microscopy images of XIAP (red), DAPI (blue), and GFAP or Iba1 (green) immunostaining in the dentate gyrus region of the hippocampus (**d**). The boxed area is shown in higher magnification. Bars represent means ± standard error of mean (SEM). **p < 0.01 vs. vehicle-treated water group. ^#^*p* < 0.05 and ^##^*p* < 0.01 vs. vehicle-treated DSS group (*n* = 4–6 per group)

As shown in Fig. 10, two-way ANOVA showed significant main effects for treatment [*F* (1, 20) = 11.92, *p* = 0.0025] but no interaction for XIAP [group × treatment: *F* (1, 20) = 1.23, *p* = 0.2805] (Fig. 10b). Thus, we focused our analysis on the effects of treatment. EF-2001 significantly increased XIAP in the hippocampus of DSS-treated mice. Cleaved caspase-3 in the hippocampus of DSS-treated mice was significantly increased compared with controls, while EF-2001 treatment significantly decreased cleaved caspase-3 levels in DSS-treated mice [Fig. 10c, two-way ANOVA, group: *F* (1, 15) = 7.46, *p* = 0.0155, treatment: *F* (1, 15) = 15.51, *p* = 0.0013, group × treatment: *F* (1, 15) = 13.91, *p* = 0.0020]. Moreover, to determine the cell types that are involved in the production of XIAP, dual immunofluorescence staining for XIAP was performed in conjunction with cell-specific markers, such as GFAP and Iba-1. XIAP was localized in the microglia (Fig. 10d).

## Discussion

Patients with IBD have higher rates of psychiatric disorders, such as depression and anxiety; however, the mechanisms underlying a link between intestinal inflammation and depressive-like symptoms are largely unknown. In this study, we investigated the effect of EF-2001 in IBD-like physiological changes and depressive-like behavior in DSS-treated mice. Chronic administration of EF-2001 prevented such changes. In addition, EF-2001 attenuated the increase of inflammatory cytokines in the rectum and hippocampus, attenuated the reduction of neurogenesis in the hippocampus, and facilitated the NFκB p65/XIAP pathway in the hippocampus of DSS-treated mice. This is the first report that the antidepressant effect of EF-2001 may involve hippocampal neuroprotection via decreased inflammatory cytokine expression in the rectum and hippocampus as well as apoptotic cell death regulation via inhibition of caspase-3 activity through facilitation of the NFκB p65/XIAP pathway in the hippocampus.

IBD, including Crohn’s disease and ulcerative colitis, is a chronic relapsing condition characterized by intestinal damage (barrier disruption, altered microbiota) and high levels of inflammation such as elevated inflammatory cytokines [43, 46, 47]. Cytokines induce extensive inflammation in the colon, which has a negative impact on epithelial cells, resident and recruited immune cells, and stromal cells [44]. Specifically, inflammation can cause damage to epithelial cells, and activate and recruit immune and stromal cells, ultimately leading to non-resolving chronic inflammation and the development of IBD [44]. Moreover, during acute or chronic inflammation, inflammatory cytokines can induce the development of depression [48, 49]. Clinical studies have reported that patients with IBD often exhibit obsessive–compulsive disorder, panic disorder, depression, and anxiety [4–7]. DSS treatment induces colonic epithelial cell lesions and intestinal inflammation, including elevated inflammatory cytokines, via epithelial cell toxicity, increased intestinal permeability, and macrophage activation [9]. It has been suggested that *E. faecalis* improves colitis by increasing interleukin-10 (IL-10), a factor that inhibits cytokine synthesis, in colonic epithelial cells [50, 51]. The present study showed that EF-2001 reduced inflammatory cytokines in the rectum (Fig. 5). This effect was similar to that of steroids, which are commonly prescribed for treating IBD [52]. Recently, other researchers have reported that EF-2001 protects dinitrobenzene sulfonic acid-induced colitis, a chemically induced colitis model, via a decrease in inflammatory cytokines [30]. Thus, the anti-inflammatory effect of EF-2001 may also be related to reduced inflammatory cytokines. Peripheral inflammation is a risk factor for developing mood or psychotic disorders, such as depression [53–56], and may affect hippocampal neurogenesis, including the proliferation, differentiation, and survival of newborn neurons [54, 55]. Adult neurogenesis occurs in two main regions of the brain, one of which is the subgranular zone of the DG [57, 58]. In the present study, we observed that DSS treatment significantly increased TNF-α and IL-6 levels in the rectum and hippocampus. A previous study has reported that DSS treatment significantly increases rectal TNF-α and IL-6 levels [59]. Interestingly, hippocampal TNF-α and IL-6 mRNA in DSS-treated mice were unchanged compared with controls (Fig. 6). Depression is closely associated with altered inflammation [16], manifested by increased expression of inflammatory cytokines such as TNF-α and IL-6 [17]. Neuroinflammatory factors, such as TNF-α and IL-6, can negatively affect many stages of neurogenesis in the adult mammalian brain, including the proliferation, differentiation, and survival of newborn neurons [18–20, 54, 55]. Therefore, cytokine-induced reduction of neurogenesis might establish a key link between inflammation and depression. In this study, DSS-treated mice showed a significant decrease in neurogenesis in the DG, consistent with a previous study [12]. Likewise, DeCarolis and Eisch reported a reduction in neurogenesis in the hippocampus of patients with depression [60]. These findings suggest that DSS-induced depressive-like behavior may be associated with the reduction of neurogenesis in the DG via the release of inflammatory cytokines derived from peripheral inflammation. Moreover, several studies have suggested that antidepressant effects are critically dependent on intact adult neurogenesis and may be mediated by the enhancement of neurogenesis in the hippocampal DG [21, 23, 24]. We observed that administration of EF-2001 significantly attenuated the enhancements of rectal and hippocampal inflammation and reduction of newborn neurons in the hippocampus of DSS-treated mice. Therefore, we suggest that the antidepressant effect and enhanced neurogenesis observed upon EF-2001 administration are partly independent effects, resulting from the EF-2001-mediated reduction of peripheral inflammation. Although the mechanism by which pro-inflammatory cytokines reduce neurogenesis is not fully understood, we believe that inflammatory cytokines in peripheral tissue might be key mediators of this process.

In human neutrophils, the activation of NFκB p65 seems to control spontaneous apoptosis and anti-apoptotic effects. Unexpectedly, we found that the activation of NFκB p65-positive cells were increased by EF-2001 administration in water- and DSS-treated groups compared with control groups. Moreover, we found that activation of NFκB p65 was localized to hippocampal astrocytes and microglia; however, the mechanisms underlying this activation remain unclear. We hypothesized that β-glucan may be associated with activation of NFκB p65 via TLR2. β-Glucan activates TLR2 and increases TLR2 expression via NFκB p65 activation [61–63]. Interestingly, we found that hippocampal TLR2 was activated by EF-2001, which was localized in all cell types, including immature and mature neurons, astrocytes, and microglia (Fig. 9). We did not assess the migration of EF-2001 or β-glucan into the brain; we will examine them in a future study. Modulation of this pathway most likely regulates the balance between pro- and anti-apoptotic factors [64], thus affecting neutrophil survival. In this study, EF-2001 attenuated DSS-induced neuroinflammation and we hypothesized that NFκB p65 may play a role in anti-apoptosis. The activation of NFκB p65 inhibits apoptosis via a mechanism involving upregulation of various anti-apoptotic genes, such as cellular FLICE-inhibitory protein, Bcl-xL, and XIAP [26, 27]. XIAP, a key member of the inhibitors of apoptosis protein family, can inhibit apoptosis by directly binding to the initiator caspases: caspase-3, -7, and -9 [65]. Moreover, hippocampal XIAP regulates synaptic plasticity, which is associated with the development of depression [66, 67]. In this study, XIAP was significantly increased by EF-2001. Further, XIAP was localized in hippocampal microglia. Moreover, cleaved caspase-3, which is crucial in the process of apoptosis and contributes to the irreversible stage of apoptosis [68], was significantly increased in the hippocampus of DSS-treated mice. In contrast, EF-2001 suppressed the increased levels of cleaved caspase-3 in the hippocampus of DSS-treated mice. These results suggested that EF-2001 might partly modulate apoptosis via regulation of the microglial NFκB p65/XIAP pathway and caspase-3 in the hippocampus of DSS-treated mice.

**Fig. 11:**
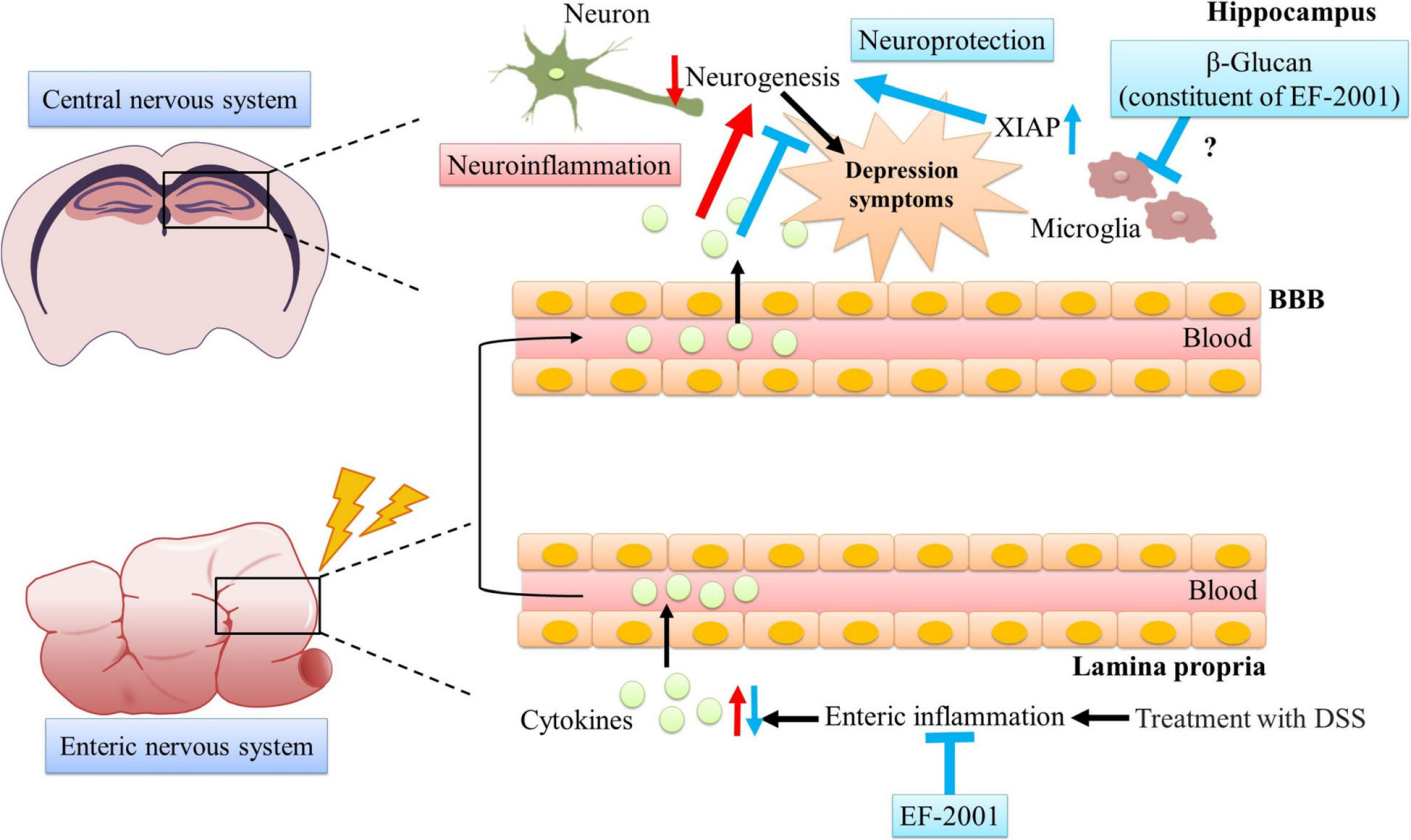
Schematic representation of the anti-inflammatory and antidepressant effects of *Enterococcus faecalis* 2001 (EF-2001). The inflammatory cytokines derived from peripheral inflammation induced by treatment with dextran sulfate sodium may induce depressive-like symptoms via reduction of neurogenesis in the hippocampus (red arrow). The inhibition of inflammatory cytokines in the rectum may explain the anti-inflammatory and antidepressant effects of EF-2001 (blue arrow). Moreover, the antidepressant effect may be caused by neuroprotection via the modulation of XIAP in the hippocampus (blue arrow)

As summarized in Fig. 11, EF-2001 had anti-inflammatory and antidepressant effects in DSS-treated mice, and hippocampal neuroprotection is a key factor mediating the antidepressant effect of EF-2001. Other researchers have reported that administration of *Lactobacillus* strains had antidepressant effects and enhanced neurogenesis in the hippocampus via the vagus nerve [69, 70]. Hence, further extensive experiments examining the relationship between the vagus nerve and effects of EF-2001 will be presented in a subsequent paper.

## Conclusions

The present study showed that DSS-treated mice exhibited IBD-like physiological changes and depressive-like behaviors (Figs. 2 and 3). This result was consistent with previous studies [11, 12]. In this study, we evaluated the predictive validity of the IBD animal model by using a classic antidepressant drug, Imi, or a steroid, Dex. Administration of Imi significantly improved depressive-like behavior, whereas Dex significantly improved both IBD-like peripheral symptoms and depressive-like behavior. These results demonstrated that DSS-treated mice provided a model of IBD with depression.

Our results indicate that EF-2001 attenuated IBD-like symptoms and depressive-like behavior in DSS-treated mice. EF-2001 prevented DSS-induced colitis and the mechanism may also involve the suppression of inflammatory cytokines in the rectum. The antidepressant effect of EF-2001 may involve neuroprotection in the hippocampus via decreased TNF-α and IL-6 expression in the rectum and hippocampus and facilitation of the NFκB p65 pathway in the hippocampus. This process is likely mediated by modulation of XIAP, which is involved in the regulation of apoptotic cell death via caspase-3 activity. Moreover, our findings suggest a close relationship between IBD and depression.

## Data Availability

The datasets used and/or analyzed in the current study are available from the corresponding author on reasonable request.

## Abbreviations

ANOVA: Analysis of variance
BDNF: Brain-derived neurotrophic factor
bp: Base pair
BrdU: 5-bromo-2′-deoxyuridine
BRM: Biological response modifier
DAI: Disease activity index
DCX: Doublecortin
Dex: Dexamethasone
DG: Dentate gyrus
DSS: Dextran sulfate sodium
EF-2001: *Enterococcus faecalis* 2001
GFAP: Glial fibrillary acidic protein
HE: Hematoxylin and eosin
i.p: Intraperitoneally
Iba1: Ionized calcium-binding adaptor molecule 1
IBD: Inflammatory bowel disease
IL-6: Interleukin-6
Imi: Imipramine
NFκB: Nuclear factor-kappa B
p.o.: Per os
PBS: Phosphate-buffered saline
PBSGT: PBS containing 1% normal goat serum and 0.3% Triton X-100
SEM: Standard error of the mean
TBP: TATA-binding protein
TLR2: Toll-like receptor 2
TNF-α: Tumor necrosis factor-α
XIAP: X-linked inhibitor of apoptosis protein
